# Anaphylaxis-related deaths in Ontario: a retrospective review of cases from 1986 to 2011

**DOI:** 10.1186/1710-1492-10-38

**Published:** 2014-07-22

**Authors:** Ya Sophia Xu, Monika Kastner, Laurie Harada, Anna Xu, Jane Salter, Susan Waserman

**Affiliations:** 1 Department of Allergy & Immunology, McMaster University, Hamilton, ON L8N 4 K1, Canada; 2 Li Ka Shing Knowledge Institute of St. Michael’s Hospital, Toronto, ON M5B 1 W8, Canada; 3 Anaphylaxis Canada, Toronto, ON M2J 5B4, Canada; 4 Department of Physiology and Pharmacology, Western University, London, ON N6A 5C1, Canada; 5 Independent MD, Toronto, Canada; 6 Department of Medicine, McMaster University, Hamilton, ON L8S 4 K1, Canada

**Keywords:** Anaphylaxis, Severe allergic reaction, Anaphylaxis mortality, Food allergy, Medication allergy, Adverse drug reaction, Venom allergy, Insect sting allergy, Iatrogenic anaphylaxis

## Abstract

**Background:**

Examining deaths caused by anaphylaxis may help identify factors that may decrease the risk of these unfortunate events. However, information on fatal anaphylaxis is limited. The objectives of our study were to examine all cases of fatal anaphylaxis in Ontario to determine cause of death, associated features, co factors and trends in mortality. The identification of these factors is important for developing effective strategies to overcome gaps in monitoring and treatment of patients with food allergies and risk for anaphylaxis.

**Methods:**

This was a retrospective case-series analysis of all causes of anaphylaxis-related deaths using data from the Ontario Coroner’s database between 1986 and 2011. Quantitative data (e.g. demographic) were analyzed using descriptive statistics and frequency analysis using SPSS. Qualitative data were analyzed using content analysis of grounded theory methodology.

**Results:**

We found 92 deaths in the last 26 years related to anaphylaxis. Causes of death, in order of decreasing frequency, included food (40 cases), insect venom (30 cases), iatrogenic (16 cases), and idiopathic (6 cases). Overall, there appears to be a decline in the frequency of food related deaths, but an increase in iatrogenic causes of fatalities. We found factors associated with fatal anaphylaxis included: delayed epinephrine administration, asthma, allergy to peanut, food ingestion outside the home, and teenagers with food allergies.

**Conclusions:**

Our findings indicate the need to improve epinephrine auto-injector use in acute reactions, particularly for teens and asthmatics with food allergies. In addition, education can be improved among food service workers and food industry in order to help food allergic patients avoid potentially fatal allergens. The increasing trend in iatrogenic related anaphylaxis is concerning, and requires monitoring and more investigation.

## Background

Examining deaths caused by anaphylaxis may help identify factors that may decrease the risk of these unfortunate events. However, information on fatal anaphylaxis is limited. Published data examining anaphylaxis deaths from all causes exist only from the United Kingdom and Australia [1,2]; and only one report has investigated food allergy fatalities, which was published in the United States [3,4]. The UK data was collected from a dedicated registry of anaphylaxis deaths, which examined 124 deaths from 1992 to 1998, and 48 deaths from all causes between 1999 and 2006 [1,5]. This study found that epinephrine and poor asthma control were risk factors for fatalities [1]. The Australian study used a national database of mortality and hospital admissions to identify changing trends in anaphylaxis death causes [2]. They evaluated 112 deaths between 1997 and 2005, and found no increase in death rates for food-induced anaphylaxis, despite an increase in food-induced anaphylaxis admissions. The US data came from a voluntary registry maintained by the American Academy of Allergy, Asthma & Immunology and the Food Allergy and Anaphylaxis Network [3,4]. These studies investigated food-related deaths between 1994 and 2006, and found that 64 of these deaths were due to a lack of timely access to epinephrine. Additionally, fatalities were more common in teens and young adults and in those with peanut and tree nut allergies [3,4]. These studies show the persistence of existing gaps in food allergy and anaphylaxis management, but may also highlight potentially modifiable risk factors that are associated with fatal anaphylaxis. The identification of these factors is important for developing effective strategies to overcome gaps in monitoring and treatment of patients with food allergies and risk for anaphylaxis. There is no published Canadian data on anaphylaxis mortality. The objectives of our study were to examine all cases of fatal anaphylaxis in Ontario for cause and trends of death between 1986 and 2011, and to identify factors that may be associated with fatality.

## Methods

We conducted a retrospective case-series analysis of anaphylaxis-related deaths from January 2000 to November 2011 using the Ontario Coroner’s database. We also included Ontario Coroner’s data from 1986 to 2000 that was previously collected by Anaphylaxis Canada [6]. The study was approved by the McMaster University Health Sciences Research Ethics Board. The Ontario Coroner’s database contains deaths that are “sudden and/or unexpected” and if “the deceased died as a result of misadventure” [7]. The Coroner’s case file includes documentation of the cause(s) of death, and details of the fatal event as reported by first responders, police and hospital staff. Some cases also include details of laboratory investigations pre and post mortem, and autopsy findings.

Cases were identified from coroner’s reports of deaths from January 1986 to November 2011 using the following terms: “Allergic Reaction”, “Anaphylaxis”, “Adverse Drug Reaction”, “Insect sting”, and “Animal/Snake bites.” These search terms were based on those used by Anaphylaxis Canada in their 1986-2000 analysis of the Coroner’s database. We added the terms: “Animal/Snake bites” to determine if additional cases could be identified. The identification code “Anaphylaxis” was not implemented in the Coroner’s electronic database until the beginning of 2003, so cases of anaphylaxis prior to 2003 were identified by a manual search of this term.

Our primary outcomes were allergy related death (e.g., food, insect), and the specific causes of these deaths. Secondary outcomes were co factors for allergy related death, such as previous allergic reaction(s), asthma, use of beta blockers, and access to epinephrine auto-injectors. We also documented patient demographics (age, sex), any available documentation of the incident leading up to the fatal reaction, and the medical management during the acute reaction.

Quantitative data (e.g. demographic and all other dichotomous data) were analyzed using descriptive statistics and frequency analysis and chi-square tests for continuous data using SPSS. We performed regression analysis to estimate whether mortality trends were statistically significant. Qualitative data (e.g. description of incident leading up to the fatal reaction) were analyzed using content analysis of grounded theory methodology [8,9].

## Results

From 1986 to 2011, there were 92 deaths in Ontario attributed to anaphylaxis (mean age 46.5 years [age range 9 to 86 years], 57% men). The characteristics of the deceased cases are in Table 1. Most cases were adults (N = 79). Of 12 paediatric cases, 10 were teenagers (defined as 11-18 years of age). The last pediatric death occurred in 2003.

**Table 1:** Characteristics of deceased persons (N = 92)

**Characteristic**	**N (%)**
Adults (>18 years of age; mean age 52)	79 (85.9)
Pediatric cases (≤18 years of age)	12 (13)
Teenagers (11-18 years)	10 (10.9)
All males	52 (57)
Mean age (years)	46.5
Age range	9 to 86
**Co-factors**	
Previous allergic reaction to suspected fatal allergen	47 (51.0)
Reported to have asthma	26 (28.3)
Other	5 (5.4)
On beta blocker and ACE-I	2 (2)
On beta blocker alone	3 (3)
On ACE-I alone	5 (5)
**Epinephrine data**	
Epinephrine prescribed or advised	19 (21).
Epinephrine nearby at the time of reaction	10 (11)
Epinephrine received prior to cardiac arrest	21 (23)
Epinephrine given subcutaneous	5 (5)

### Causes of anaphylaxis related death

Overall, the most common cause of allergy related death was to food (40 cases; 43%), followed by insect venom (30 cases; 33%) and iatrogenic (16 cases; 17%). Six cases of anaphylaxis did not have an identified cause (Table 2).

**Table 2:** Causes of death (N = 92)

**Cause of death**	**Number of deceased persons (%)**	**Average age (Age range)**	**Male (%)**
**Food**	40 (43)	32 (9-78)	19 (47.5)
Peanut	16 (17)	-	-
Tree nuts	6 (6.5)	-	-
Seafood	4 (4)	-	-
Milk	1 (2.5)	-	-
Other foods	7 (18)	-	-
Unknown food	6 (7)		-
**Insect venom**	30 (33)	54 (25-77)	24 (80)
**Medication**	16 (17)	65 (39-86)	6 (37.5)
Antibiotic (Penicillin, Cephalosporin, Septra)	7 (8)	-	-
Radiocontrast dye	4 (4)	-	-
**Unclear cause** (more than one culprit, or no suspected allergen)	6 (7)	64 (51-83)	3 (50)

The most common food allergen was peanut, which was responsible for 16 fatalities (17%). Six deaths were attributed to tree nuts (6.5%), 4 to seafood (4.3%), and 1 to milk (1%). An additional 7 deaths were attributed to other types of food allergens (peach, berry, grape juice, beer, tomato, sesame seed, and MSG). There were 6 deaths consistent with a food-related reaction, but the culprit food was unknown or unclear. In these 6 cases, food was believed to be a trigger because features of the reaction fit with anaphylaxis, but multiple foods were ingested, so the culprit food(s) could not be identified with certainty.

The main causes of medication related anaphylaxis were antibiotics (7 cases), and radio contrast dye (4 cases). Among antibiotic related deaths, 3 cases were related to penicillin allergy, 2 to trimethoprim sulfamethoxazole, and 2 to cephalosporins. The 5 remaining deaths were related to Fentanyl, ASA, Protamine, Pindolol, and Bupropion.

Of the 92 people who died from anaphylaxis, 47 cases (51%) had a known or suspected allergy to the fatal allergen. Among the 40 deaths related to food allergy, 34 cases (85%) were reported to have known or suspected allergy to the culprit food. In contrast, only 11 people (37%) who died from insect stings and 1 person (6%) who died from medication allergy had known or suspected allergy to the fatal allergen. Data was not available on prior reactions to the fatal allergen in 11 cases of patients who died from medication related allergy, 19 cases from venom allergy, and 6 cases from food fatalities.

### Features associated with anaphylaxis

#### Delayed and Lack of Epinephrine administration

Of the 47 people with a known or suspected allergy, 18 were prescribed an epinephrine auto-injector (38%). Of these, only 10 people (57%) carried an auto-injector at the time of the reaction. There was inadequate information on the specific time between onset of reaction and when epinephrine was given in the Corners’ report. We found that 21 of the 92 cases (23%) received epinephrine prior to cardiac arrest. In two cases that did not have their own prescription of epinephrine, one used an auto-injector from another patient, and the other received it from a pharmacy. Furthermore, in the 7 iatrogenic reactions that occurred in the hospital, epinephrine was given prior to cardiac arrest in only 3 cases (42%). Five people received epinephrine subcutaneously, and 1 received an expired epinephrine auto-injector.

#### Asthma status

Asthma diagnosis and control was examined as potential contributors to severe anaphylaxis. We identified 26 people with a diagnosis of asthma (28%); 2 cases were reported not to have asthma. Among the 26 diagnosed cases, 8 people (9%) were reported to have poorly controlled asthma, and 3 people had mild or controlled asthma. The status of asthma control was not reported in 15 of the 26 asthma cases (58%). Since 64 of the fatality cases (70%) did not have asthma status documented in the coroner’s database, we could not definitively determine if they had asthma or not.

#### Medication

Of cases that had documented intake of medications prior to death, two people were reported as having taken both angiotensin-converting enzyme inhibitors (ACEIs) and beta blockers, 3 people had taken beta blockers alone, and 5 had taken ACEIs alone.

#### Location of fatal reaction

Of the 40 fatalities caused by food allergies, 14 cases occurred at home. The remainder 24 cases occurred outside the home including at restaurants or a wedding (n = 15), at school (n = 4), camp (n = 3), and at another person’s home (n = 2). Location of reaction was not reported in 2 cases. Among the 34 cases with a previous food related reaction, 3 people knowingly ingested the allergen (one case was a suicide), and in 7 cases, people failed to read labels restaurant menus or ask about ingredients. An almost equal number of drug reactions occurred at home (n = 8) and the hospital (n = 7). One death occurred at a long term care facility. Fatal drug reactions that occurred at home (n = 8) were most commonly caused by antibiotics (63%). One patient had a previous reaction to the responsible antibiotic. The most common culprit in hospital reactions (n = 7) was radiocontrast dye (4 cases), for which there was no prior allergy reported in all 4 cases. In 22 cases, the Coroner’s report did not include enough information to determine the location of the fatal reaction.

### Trends in causes of death

**Figure 1:**
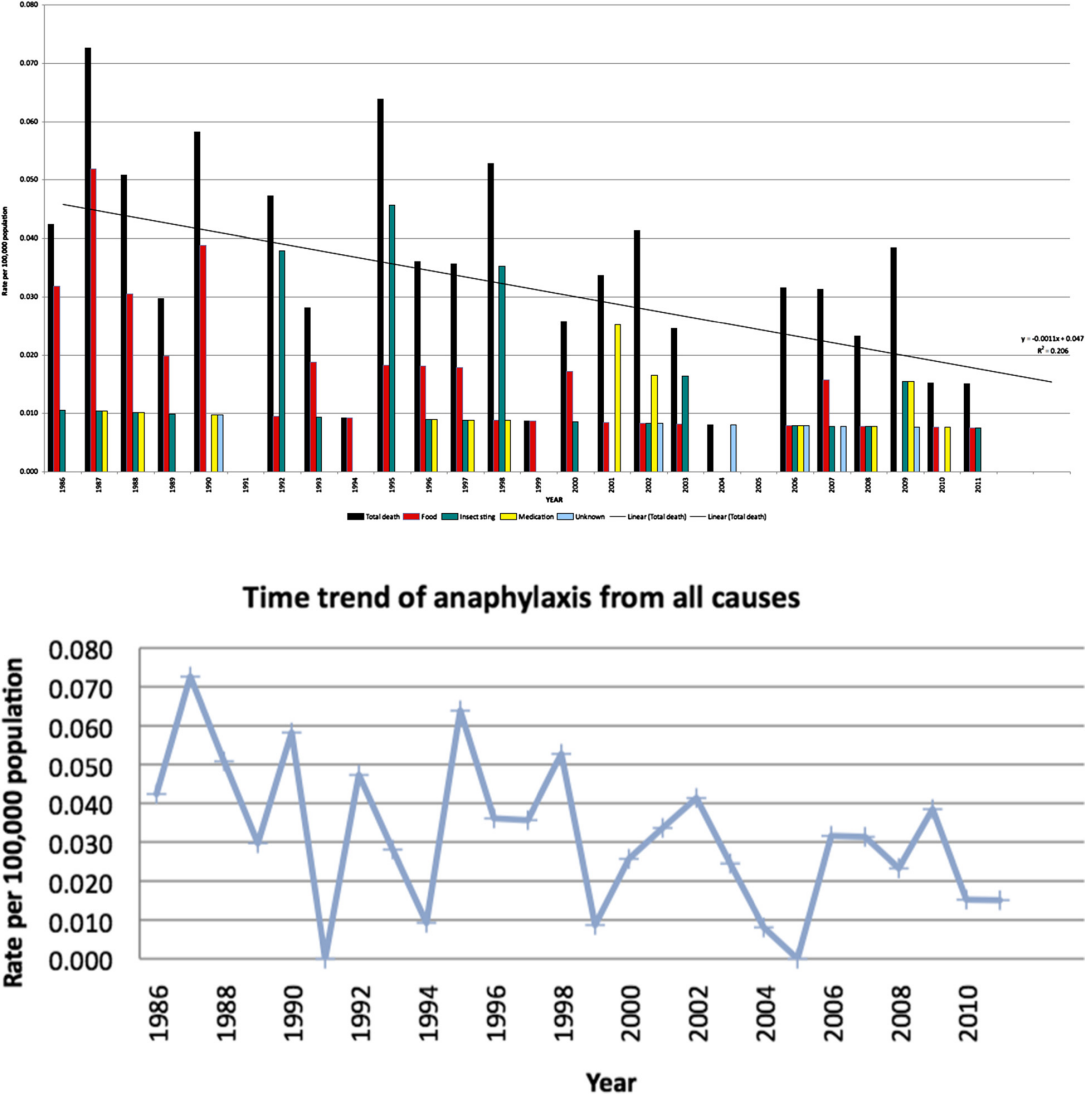
Time trend for anaphylaxis death in Ontario from 1986 to 2011.

**Figure 2:**
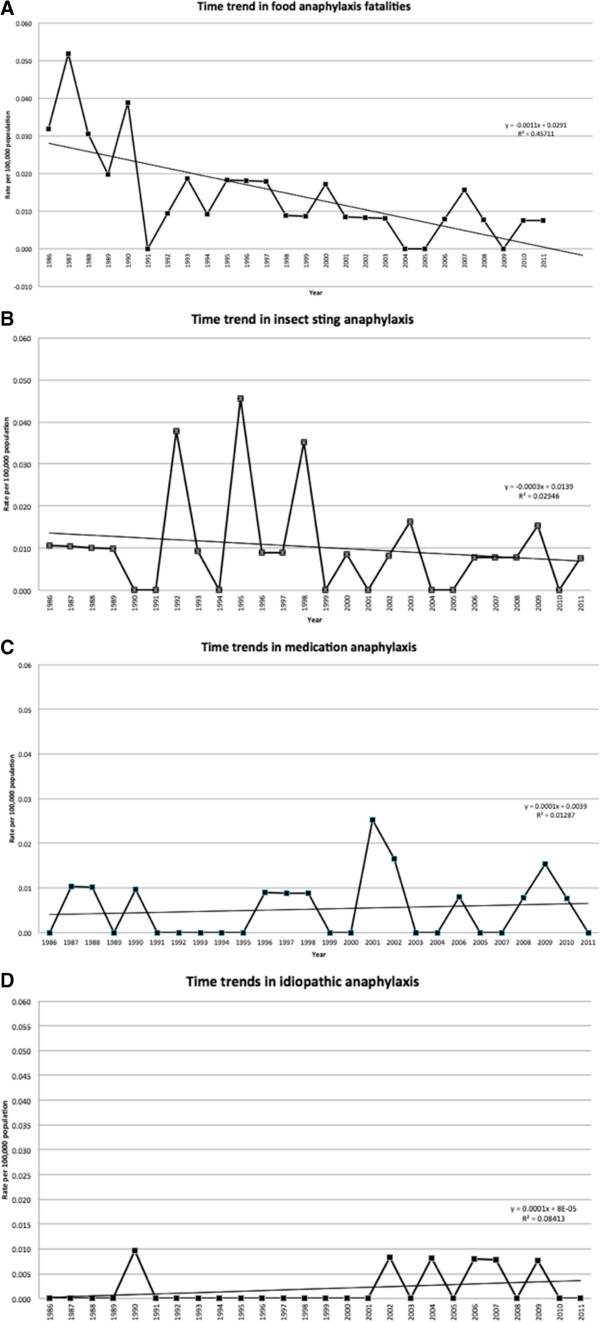
**Time trends for food-induced and non–food–induced anaphylaxis fatalities. A**. Time trend in food anaphylaxis fatalities. **B**. Time trend in insect sting anaphylaxis. **C**. Time trends in medication anaphylaxis. **D**. Time trends in idiopathic anaphylaxis.

Our data indicates a decline in all causes of anaphylaxis related mortality (see Figure 1). This trend was significant (R^2^ = 0.206; p = 0.02). Between the two time periods we investigated (1986-1998 and 1999-2011) there has been a decline from 55 deaths to 37 deaths. Furthermore, fatalities due to food declined from 28 to 12 cases, with those related to peanut and tree nuts decreasing from 16 to 6 cases. Similarly, stinging insect venom as a cause declined from 20 to 10 cases. In contrast, fatalities have increased for medication related (from 6 to 10) and unknown causes (from 1 to 5). These trends of death by cause can also be seen in Figure 2. Among the 16 iatrogenic related deaths, there appears to be an increase in reactions in hospitals in recent years: there were 5 cases of iatrogenic death between 2001 to 2011, compared to 3 cases between 1986 to 2000. Radiocontrast dye related fatalities represented the majority of hospital-based reactions, which have increased from 1 case between 1986 to 2001 to 3 cases between 2002 to 2011.

### Autopsy findings

Autopsies were performed in 65 of the documented deaths (71%). In 2 cases, autopsy was performed after the embalming process. The most common reported findings on autopsy were pulmonary congestion and/or edema (60%), upper airway edema (46%), mucus plugging of bronchi and/or hyperinflation (17%), cerebral edema (15%), congestion of liver and/or spleen (14%), petechial haemorrhage (14%), and signs of vomiting (8%). Amongst the 30 venom related deaths (33%), evidence of insect sting was found in 12% of cases. Severe edema was found in 46% of those with upper airway edema and 11% with lower airway findings (Table 3).

**Table 3:** Autopsy findings (N = 65)

**Autopsy finding**	**Number affected (%)**
Pulmonary congestion and/or edema	39 (60)
*Severe edema*	7 (11)
*Mild edema*	5 (8)
Upper airway edema (from lips to larynx)	30 (46)
*Severe edema*	11 (17)
*Mild*	5 (8)
Mucus plugging of bronchi and/or hyperinflation	11 (17)
Evidence of begin stung by insect	8 (12)
*Skin showing site of sting*	7 (11)
*Hymenoptera found in vomit*	1 (2)
Acute GI findings	
*Congestion of liver and/or spleen*	9 (14)
*Signs of vomiting (e.g. vomit in airway)*	5 (8)
*Gastric hemorrhage*	3 (5)
CNS findings	
*Cerebral edema*	10 (15)
*Cerebral ischemia*	5 (8)
Petechial hemorrhage	9 (14)
*Lungs*	3 (5)
*Eye*	2 (3)
*Skin*	2 (3)
*Multi-organ*	2 (3)
Skin edema	5 (8)
Conjunctiva edema	4 (6)
Acute cardiovascular findings	
*Acute MI*	3 (5)
*Acute myocardium congestion*	1 (2)

## Discussion

Our study, which spanned 26 years have shown that the total number of anaphylaxis fatalities has declined, despite population growth in Ontario [10]. The biggest declines were observed in food and insect venom related deaths. In contrast, there was a slight increase in anaphylaxis deaths related to medication allergy and unknown or idiopathic causes. Similar trends were seen in Australian data, where mortality rates from insect sting-induced anaphylaxis decreased, while drug-induced anaphylaxis fatalities were reported to be on the rise [2].

The increase in deaths from iatrogenic allergy may be related to increasing exposure of medications and procedure, such as radiocontrast dye that can lead to reaction with first time exposures. We also found that epinephrine administration was delayed during reactions that occurred in hospital. This indicates that health care providers may require more education to more appropriately recognize and manage acute allergic reactions. Monitoring and further study of iatrogenic related allergies are needed.

The overall decline in food-related fatalities may reflect increasing public awareness and improved education in managing food allergy by patients with allergies and their families. This may in part be due to the public health legislation of “Sabrina’s law”, which became effective on January 1, 2006, after the tragic death of a teenager, Sabrina Shannon, who died of a fatal food related reaction after an accidental ingestion while at school. The law requires that every school board in Ontario establish and maintain an anaphylaxis policy including the development of individual plans for pupils at risk for anaphylaxis [11].

Our findings also indicated inadequate avoidance of allergens and treatment of acute allergic reactions. Among the deaths related to food allergies, 85% of cases had known or suspected allergy to the fatal allergen. This trend was also reported in a US study, where all but 1 of 32 subjects was known to have food allergy before the fatal event [3]. Our study also found that only 19 of the 48 people who had a known or suspected allergy (40%) were prescribed an epinephrine auto-injector, only 10 of the 19 people (53%) had their auto-injector with them at the time of the reaction, and 22 of the 92 fatalities (24%) received epinephrine prior to cardiac arrest. Five people received epinephrine subcutaneously, and 1 received an expired auto-injector. Similar trends were seen in the study by Pumphery et al, which analyzed 48 food related deaths from 1999 to 2006. This study found that epinephrine auto-injector pens had been given only to 19 of those deceased (40%), were used correctly by 9 of 19 people (47%), and 2 people had used expired pens [5]. Furthermore, four people did not carry the pen at the time of the reaction, 5 used the pen too late, and one person misused it [5]. The persistence of findings related to problems in using an epinephrine auto-injector during acute reactions indicate that more patient education is needed, and perhaps better public access to these devices such as providing epinephrine auto-injectors in public places much like automatic defibrillators.

Asthma as a risk factor for anaphylaxis-related mortality has been identified in several studies [5,12]. In our data, we found that 28% of the deceased were reported to have asthma, which is higher than what has been reported in the general population (8-10%) [13]. However, it is likely that this rate was higher in our study. Asthma status of 64 cases (70%) was not reported, and we were not able to verify if they had asthma or not. Additionally, we did not examine anaphylaxis related deaths, so it is possible that some of these cases may have been misfiled under asthmatic deaths, which likely underestimated the number of asthmatics who died of anaphylaxis.

Allergy to peanuts was a common cause of fatality as it accounted for 16 food-related fatalities in our study (40%). Higher proportion of peanut related deaths have also been reported in US studies, where peanuts accounted for 53%-64% of the fatalities [3,4].

Of those who died of food allergies in our study, all but 3 cases unknowingly ingested the food allergen. Since 85% had known or suspected allergy to the fatal allergen, it is possible that they did not inquire about allergens or were given incorrect information. A recent survey of 1454 food allergic patients investigating inadvertent exposures, found that 47% of respondents attributed the event to inappropriate labelling, 28.6% to failure to read a food label, and 8.3% to ignoring a precautionary statement [14]. More consistent labelling of foods in plain language, as well as providing continuous patient education on vigilance around allergen avoidance may reduce accidental ingestions and food-related fatalities.

We also found that exposure to fatal foods occurred slightly more common outside the home (24 of 40 cases), especially in restaurants and food courts (15 cases). Similar trends were seen in food related deaths in a US study of 32 fatal reactions, where 81% of deaths occurred outside of the home [4]. A recent Canadian study of 1411 children with peanut allergy also found that 61% of inadvertent exposures had occurred outside of the home [14]. These higher occurrence of food related fatalities outside of the home point to the need to expand educational efforts on food allergen avoidance to the restaurant and food service industry, perhaps as part of work place orientation. Appropriate use of epinephrine auto-injector use may also be improved by making them available in malls, restaurants and other public areas where food is consumed.

Our autopsy results were consistent with previous reports of anaphylaxis autopsy features, which indicated that the most common features were pulmonary edema and upper airway edema [15,16]. Pulmonary congestion and/or edema are not specific to anaphylaxis. Rather, they reflect cardiogenic shock, which is the end point of various causes of death. Upper airway edema, mucus plugging of bronchi and/or hyperinflation, and petechial haemorrhage are more suggestive of an allergic reaction and respiratory distress. However the diagnosis of anaphylaxis remains primarily a clinical one.

The major strengths of our study are that we collected data on anaphylaxis deaths over a 26-year period, and we are the first to perform this investigation in Ontario. Our study has some limitations. We may have underestimated the number of deaths related to anaphylaxis by not including cases coded under death caused by asthma. Preliminary search for asthma as a “death factor” returned hundreds of cases, so the investigation of these would have been beyond the scope of our study. Furthermore, we found that most of the coroners’ reports were incomplete, which may have precluded a definitive identification of asthma diagnosis. We also may not have identified all cases of anaphylaxis prior to 2003 since the code for anaphylaxis as a cause of death has not yet been introduced. However, we manually searched the database prior to 2003 in an attempt to identify all anaphylaxis-related deaths. Another limitation is that we did not include controls in our analysis. However, our objective was to investigate and describe a series of anaphylaxis deaths rather than to make comparisons and investigate possible associations or causation. Lastly, some important details about the deaths were not recorded in the Coroner’s database, so we were not able to determine details surrounding the fatality such as previous asthma control, the exact time that epinephrine was administered in relation to onset of reaction, as well the route and location of epinephrine injection.

## Conclusions

Food-induced anaphylaxis death rates in Ontario appear to have declined in the past 12 years, despite increasing population growth and growing prevalence of food allergy. This suggests that public awareness and efforts by health care professionals may have had a positive effect. However, there continues to be a low rate of epinephrine prescription and administration during acute reactions. Fatalities are more common in people with food allergies, especially teens with peanut allergy. Ongoing efforts are needed to education allergic individuals and health care professionals about risks, avoidance and management.

We also found that reactions occur more frequently outside of the home, particularly in public places that serve food. This trend highlights an educational gap in the food industry. The provision of epinephrine auto-injectors in public places such as food courts may be one way to address this, similar to how cardiac defibrillators have been implemented in public places to avoid cardiac deaths. Our next steps include expanding our study to include anaphylaxis deaths in other provinces across Canada, and to develop a national registry to track mortality due to anaphylaxis aimed at identifying potentially modifiable causes and to better inform patient and public education.

## Competing interests

The authors declare that they have no competing interests.

## Authors’ contributions

YSX, SW and MK conceived and designed the study, analyzed the results and prepared the manuscript. LC, JS, MS, CJT, AC collected data from the Coroner’s database and contributed to data analysis. All authors read and approved the final manuscript.

## References

[B1] Pumphrey RS Lessons for management of anaphylaxis from a study of fatal reactions Clin Exp Allergy 2000 30 8 1144 1150 10931122 10.1046/j.1365-2222.2000.00864.x

[B2] Liew WK Williamson E Tang ML Anaphylaxis fatalities and admissions in Australia J Allergy Clin Immunol 2009 123 2 434 442 19117599 10.1016/j.jaci.2008.10.049

[B3] Bock SA MuÃ±oz-Furlong A Sampson HA Fatalities due to anaphylactic reactions to foods J Allergy Clin Immunol 2001 107 1 191 193 11150011 10.1067/mai.2001.112031

[B4] Bock SA MuÃ±oz-Furlong A Sampson HA Further fatalities caused by anaphylactic reactions to food, 2001-2006 J Allergy Clin Immunol 2007 119 4 1016 1018 17306354 10.1016/j.jaci.2006.12.622

[B5] Pumphrey RSH Gowland MH Further fatal allergic reactions to food in the United Kingdom, 1999-2006 [letter] J Allergy Clin Immunol 2007 119 1018 1019 17349682 10.1016/j.jaci.2007.01.021

[B6] Salter J Mehra S Cairns JT Sussman G Vadas P A study of 32 food-induced anaphylaxis deaths in Ontario: 1986-2000[Abstract] J Allergy Clin Immunol 2002 109 1 S181

[B7] Ontario Coroner’s act http://www.e-laws.gov.on.ca/html/statutes/english/elaws_statutes_90c37_e.htm

[B8] Strauss A Corbin J Basics of Qualitative Research: Grounded Theory Procedures and Techniques 1990 California: Sage Publications Inc

[B9] Patton MQ In Qualitative Research and Evaluation Methods 2002 3 California: Sage Publications Inc

[B10] Statistics Canada: Focus on Geography Series, 2011 Censushttp://www.statcan.gc.ca/tables-tableaux/sum-som/l01/cst01/demo02a-eng.htm

[B11] Sabrina’s Law Available at: http://www.anaphylaxis.ca/en/resources/sabrinas_law.html. Accessed on February 28, 2014

[B12] MuÃ±oz-Furlong A Weiss CC Characteristics of food-allergic patients placing them at risk for a fatal anaphylactic episode Curr Allergy Asthma Rep 2009 9 1 57 63 19063826 10.1007/s11882-009-0009-2

[B13] Center for disease control and prevention Asthma Surveillance Data http://www.cdc.gov/asthma/asthmadata.htm

[B14] Sheth S Waserman S Kagan R Alizadehfar R Primeau M Elliot S St-Pierre Y Wickett R Joseph L Harada L Dufresne C Allen M Allen M Godefroy S Clarke A Role of food labels on accidental exposures in food allergic individuals in Canada Ann Allergy Asthma Immunol 2010 104 1 60 65 20143647 10.1016/j.anai.2009.11.008

[B15] Pumphrey RS Roberts IS Postmortem findings after fatal anaphylactic reactions J Clin Pathol 2000 53 273 276 10823122 10.1136/jcp.53.4.273 PMC1731177

[B16] Greenberger PA Rotskoff BD Lifschultz B Fatal anaphylaxis: postmortem findings and associated comorbid diseases Ann Allergy Asthma Immunol 2007 98 3 252 257 17378256 10.1016/S1081-1206(10)60714-4

